# A Retrospective Analysis of Corneal Dermoid

**DOI:** 10.7759/cureus.64840

**Published:** 2024-07-18

**Authors:** Shuting Xuan, Xiaoting Pei, Zhijie Li

**Affiliations:** 1 Ophthalmology Department, Henan Provincial People’s Hospital, Zhengzhou, CHN; 2 Henan Eye Institute, Henan Provincial People’s Hospital, People’s Hospital of Henan University, First Affiliated Hospital of Zhengzhou University, Zhengzhou, CHN

**Keywords:** amniotic membrane grafting, lamellar keratoplasty, retrospective analysis, clinical characteristics, corneal dermoid

## Abstract

Background: Corneal dermoid is a congenital benign tumor and ocular malformation, often diagnosed at birth or in early childhood. Its treatment and long-term prognosis remain under-researched, necessitating further investigation.

Purpose: This study aims to investigate the epidemiological and clinical characteristics of corneal dermoid, evaluate the efficacy of different surgical methods, and identify factors influencing treatment outcomes.

Methods: A retrospective analysis was conducted on the clinical data of 58 patients treated for corneal dermoid at our hospital from 2017 to 2021. Patients' demographic information, tumor characteristics, surgical methods, and postoperative outcomes were collected and analyzed. Descriptive statistics, independent sample *t*-test, chi-square (*χ*^2^) test, and Spearman correlation analysis were used to evaluate the distribution characteristics and intergroup differences of corneal dermoid.

Results: The average age of the patients was 6.3 years, with 55.2% being male and 44.8% female. The right eye was affected in 63.8% of cases, with the temporal limbus being the most common site (75.9%). Pathological examination revealed tumors covered by squamous epithelium, containing hair follicles, sebaceous glands, adipose tissue, and fibrous tissue; some cases also had cartilage and glandular tissue. Surgical methods included corneal dermoid excision (100%), lamellar keratoplasty (37.9%), amniotic membrane grafting (31.0%), and autologous limbal stem cell transplantation (8.6%). None of the 50 followed up patients experienced tumor recurrence. Postoperative vision improved in 58.0% of patients, with more females (61.9%) experiencing visual impairment compared to males (38.1%) (*χ*²=4.711, p=0.030).

Conclusions: This study analyzed 58 corneal dermoid patients treated from 2017 to 2021, focusing on epidemiological and clinical characteristics, surgical efficacy, and treatment outcomes. It identified common pathological features and effective surgical methods, with no tumor recurrence in followed up patients. The study highlights the need for further research with larger sample sizes and longer follow-up periods.

## Introduction

Corneal dermoid is a congenital benign tumor and ocular malformation, typically identified at birth or in early childhood [1]. It manifests as ectopic dermoid tissue on the cornea or conjunctiva, characterized by white or yellowish nodules with possible hair and neovascularization on the surface [2]. Corneal dermoid can occur independently or with other systemic anomalies, such as Goldenhar syndrome and organoid nevus syndrome [3]. Although typically painless and asymptomatic, their space-occupying nature and progressive enlargement can lead to visual impairment, restricted eye movement, and facial asymmetry, adversely affecting patients' visual function and quality of life [4].

Despite progress in the diagnosis and treatment of corneal dermoid, many aspects of its pathogenesis, optimal treatment methods, and long-term prognosis remain unknown [2]. Existing literature primarily consists of case reports or small-scale studies, lacking systematic large-sample retrospective analyses [5]. There is no consensus on the choice of surgical methods or their effectiveness [6]. Understanding the epidemiology, tumor characteristics, and treatment responses of corneal dermoid is crucial for developing scientific and individualized treatment strategies.

By providing clinicians with comprehensive and accurate references, this study aims to optimize the diagnosis and treatment of corneal dermoid and improve patients' quality of life. Additionally, the results lay the foundation for future exploration of pathological mechanisms and new treatment methods for corneal dermoid.

## Materials and methods

We systematically reviewed and analyzed the clinical data of 58 patients treated for corneal dermoid at our hospital from 2017 to 2021. Our objectives were to summarize the epidemiological and clinical characteristics of corneal dermoid, evaluate the efficacy of different surgical methods and the incidence of complications, and explore factors influencing treatment outcomes. 

Study subjects

This study utilized a retrospective analysis to collect and examine clinical data of patients diagnosed with corneal dermoid who underwent surgical excision at Henan Eye Hospital from 2017 to 2021. Patients missing key information (age, gender, affected eye, surgical method) were excluded. All patients or their guardians provided informed consent. The study design and data collection adhered to the ethical principles of the Declaration of Helsinki and were approved by the Ethics Committee of Henan Provincial People's Hospital.

Patient data collection

Detailed medical records were maintained for all patients during their hospital stay, including demographic information, tumor characteristics, surgical methods, and postoperative follow-up results. Collected data included the following: (1) demographic information: age, sex, medical history, affected eye (left or right), disease duration, initial tumor discovery time, and progression; (2) symptoms: eye redness, eye pain, tearing, and vision decline; (3) tumor characteristics: location, size, corneal invasion extent, appearance, and texture; (4) surgical methods: procedures such as corneal dermoid excision, amniotic membrane grafting, lamellar keratoplasty, conjunctival sac reconstruction, and autologous limbal stem cell transplantation; and (5) postoperative outcomes: follow-up records noting recurrence, vision restoration, corneal healing, and postoperative complications.

Definitions and standards

Corneal dermoid was diagnosed by ophthalmology experts based on specific criteria. Congenital anomalies were present at birth, typically manifesting as white or yellow elevated lesions at the limbus, sometimes with hair on the surface. Lesions could invade the cornea or conjunctiva, varying in size and depth. Pathological features included coverage by keratinized stratified squamous epithelium and the presence of various tissues such as hair follicles, sebaceous glands, nerves, brain tissue, lacrimal glands, adipose tissue, fibrous connective tissue, sweat glands, cartilage, bone, and blood vessels. Classification of corneal dermoid was graded according to Mann’s classification. Grade I involved the surface of the cornea. Grade II affected the entire corneal thickness, possibly sparing Descemet's membrane and the endothelium. Grade III encompassed the entire cornea and all anterior chamber structures. Tumor locations were categorized based on quadrants. In the temporal quadrant, tumors were found in quadrant 2 or 3 for the left eye and quadrant 1 or 4 for the right eye. In the nasal quadrant, tumors were located in quadrant 1 or 4 for the left eye and quadrant 2 or 3 for the right eye. Superior tumors spanned quadrants 1 and 2, while inferior tumors spanned quadrants 3 and 4. Tumor size was measured in millimeters (mm) for maximum (horizontal) and minimum (vertical) diameters, and the area of the tumor was calculated as the product of these diameters. Corneal invasion depth was categorized as superficial for depths less than 3 mm and deep for depths of 3 mm or more. Postoperative vision was measured using a standard logarithmic vision chart, recording the smallest line of vision identified at 5 m.

Statistical analysis methods

The collected data were analyzed using Statistical Product and Service Solutions (SPSS, version 26.0; IBM SPSS Statistics for Windows, Armonk, NY). Quantitative data were expressed as mean ± standard deviation (mean ± SD) or mean (95%CI). Unpaired t-test or one-way ANOVA was used for group comparisons of normally distributed data. For skewed distributions, non-parametric tests were applied. Qualitative data were expressed as frequencies or percentages, and group comparisons were conducted using the chi-square (*χ*^2^) test. Spearman correlation analysis was used to assess the linear relationship between variables (e.g., the relationship between tumor size and age). All statistical analyses were two-sided, with a significance level set at p < 0.05. Prior to statistical analysis, stringent quality control measures were implemented for all data, including checks for completeness and handling of outliers.

## Results

General patient information

**Figure 1:**
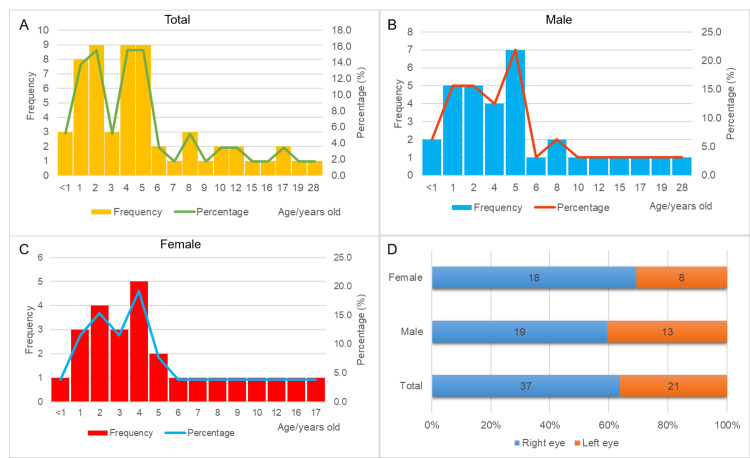
Description of the general characteristics of corneal dermoid patients (A) Age distribution of corneal dermoid patients; (B) age distribution of male patients; (C) age distribution of female patients; (D) distribution of affected eyes in corneal dermoid patients.

**Figure 2:**
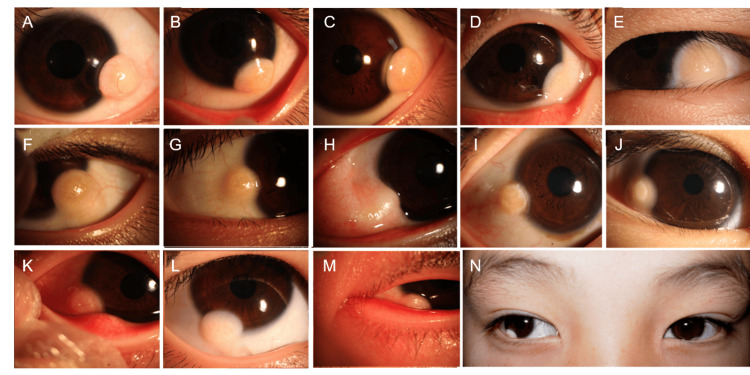
Typical clinical images in this study (A-D) Smooth corneal dermoid on the inferotemporal side of the left eye; (E) uneven corneal dermoid on the temporal side of the left eye; (F) uneven corneal dermoid on the inferotemporal side of the left eye; (G) corneal dermoid on the temporal side of the right eye; (H-L) corneal dermoid on the inferotemporal side of the right eye; (M) corneal dermoid on the lower nasal side of the left eye; (N) corneal dermoid on the inferotemporal sides of both eyes. The dermoid on the left eye is smaller and less noticeable.

This study included 58 patients who underwent corneal dermoid surgery at Henan Eye Hospital from 2017 to 2021. The patients' ages ranged from 0 to 28 years, with a mean of 6.3 ± 5.4 years and a median of five years. The interquartile range was two to nine years (Figures 1A-1C). The patients included 32 males (55.2%) and 26 females (44.8%). Thirty-seven patients (63.8%) had right eye lesions, while 21 (36.2%) had left eye lesions. For one patient with a bilateral corneal dermoid tumor, the affected eye was defined as the severe eye (Figure 2N). Among males, 19 had right eye involvement and 13 had left eye involvement. Among females, 18 had right eye involvement, and eight had left eye involvement (Figure 1D).

Reports from patients or their families indicate most corneal dermoids were discovered at birth or in early childhood without obvious triggers. Most patients were asymptomatic, with some experiencing mild photophobia, tearing, and redness, but no severe discomfort. All patients were classified as Grade I according to Mann's classification. No patients in this study had a family history of the condition. The age distribution was broad, primarily concentrated in pediatric and adolescent stages, with slightly more males than females. The higher incidence of right eye involvement suggests special attention is needed for right eye corneal dermoid in diagnosis and treatment.

Tumor characteristics

This study analyzed the tumor characteristics of 58 corneal dermoid patients. Tumor diameters ranged from 1 mm to 10 mm, with an average horizontal diameter of 4.55 mm (95% CI: 4.13-4.97 mm) and vertical diameter of 4.62 mm (95% CI: 4.23-5.00 mm) (Table 1). Most tumors measured between 3 mm and 6 mm, accounting for 75% of the total. There were no significant differences in tumor size between genders and age groups (p > 0.05) nor was there a significant correlation between age and tumor size (p > 0.05) (Table 2). However, some patients or their families reported that tumors gradually increased in size with age but without significant vision impairments.

**Table 1:** Description of tumor characteristics in patients

Variables	Total (N=58)	Male (n=32)	Female (n=26)	t/χ²	p
Tumor Size					
Horizontal diameter (mm), mean (95%CI)	4.55 (4.13-4.97)	4.40 (3.95-4.85)	4.73 (3.95-5.51)	0.783	0.437
Vertical diameter (mm), mean (95%CI)	4.62 (4.24-5.00)	4.57 (4.05-5.08)	4.67 (4.08-5.27)	0.278	0.782
Area (mm²), mean (95%CI)	22.58 (18.95-26.21)	21.37 (17.00-25.73)	23.98 (17.69-30.27)	0.737	0.464
Corneal Invasion Depth				1.444	0.230
Mean (95%CI)	2.42 (2.16-2.68)	2.26 (1.90-2.62)	2.62 (2.22-3.01)	1.373	0.176
< 3 mm, n (%)	34 (58.6)	21 (65.6)	13 (50)		
≥ 3 mm, n (%)	24 (41.4)	11 (34.4)	13 (50)		
Tumor Location				0.169	0.919
Temporal, n (%)	44 (75.9)	24 (75.0)	20 (76.9)		
Nasal, n (%)	11 (19.0)	6 (18.7)	5 (19.3)		
Inferior, n (%)	3 (5.2)	2 (6.3)	1 (3.8)		
Tumor Surface				0.592	0.442
Smooth, n (%)	15 (25.9)	7 (21.9)	8 (30.8)		
Uneven, n (%)	43 (74.1)	25 (78.1)	18 (69.2)		

**Table 2:** The correlation between age and tumor size *r_s_*: Spearman correlation coefficient

		Horizontal diameter	Vertical diameter	Area	Corneal invasion depth
Total	r_s_	0.049	0.077	0.058	0.087
	p	0.721	0.574	0.665	0.519
Male	r_s_	-0.019	0.026	0.019	0.039
	p	0.919	0.891	0.917	0.834
Female	r_s_	0.109	0.151	0.112	0.300
	p	0.597	0.462	0.586	0.136

Tumor invasion depth ranged from 0.5 mm to 5 mm, with an average of 2.42 mm (95% CI: 2.16-2.68 mm). Deeper corneal invasion (≥3 mm) was observed in 24 patients (41.4%), while shallower invasion (<3 mm) was seen in 34 patients (58.6%). There were no significant differences in corneal invasion depth between genders and age groups (p > 0.05).

Tumors were primarily located in the limbal region, with the temporal limbus being the most common site. Of the 58 patients, 44 (75.9%) had tumors at the temporal limbus (Figures 2A-2F), 11 (19.0%) at the nasal limbus (Figures 2G-2K), and three (5.2%) at the inferior limbus (Figures 2L-2M). There were no significant differences in tumor location distribution between genders and age groups (p > 0.05).

Tumors appeared white or yellow-white, mostly round or oval, and generally had uneven surfaces (Figures 2E-2F). Some tumors showed hair growth (Figure 2A) and neovascularization on the surface(Figures 2B-2C), while a few had smooth surfaces with well-defined borders (Figures 2A-2C). Based on appearance, tumors were categorized into smooth-surface (Figures 2A-2D) and uneven-surface types (Figures 2E-2F). The smooth-surface type included 15 cases (25.9%), with 12 patients aged five years or younger, while the uneven-surface type included 43 cases (74.1%). There were no significant differences between genders (p > 0.05). Tumor characteristics are summarized in Table 1.

These findings highlight the variability in tumor characteristics among corneal dermoid patients. Further detailed analysis and larger sample sizes are necessary to explore the potential impact of these characteristics on treatment outcomes and prognosis.

Pathological characteristics

**Figure 3:**
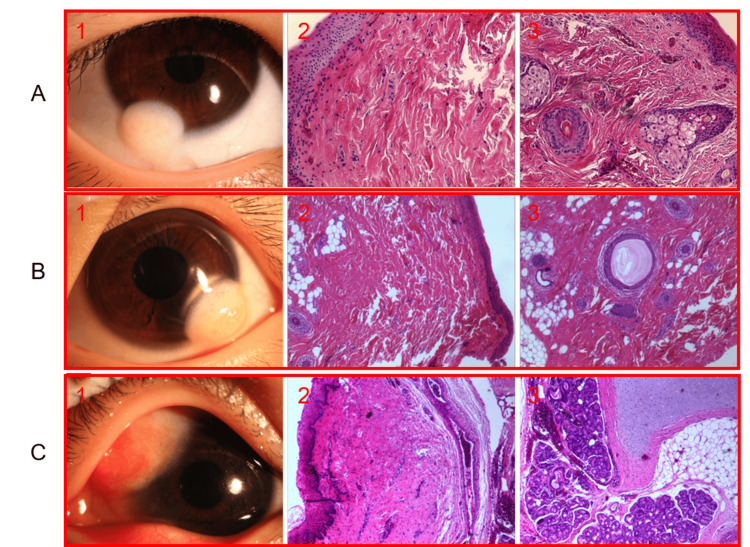
Typical clinicopathological images of corneal dermoid (A) From left to right: clinical appearance of the affected eye with pathological sampling; the submitted tissue surface covered by squamous epithelium with proliferative fibrous tissue underneath; and the dermoid mass containing skin appendages and adipose vascular tissue. (B) From left to right: clinical appearance of the affected eye with pathological sampling; the submitted tissue surface covered by squamous epithelium; and presence of adipose tissue, sebaceous gland ducts, and hair follicles within hyalinized fibrous tissue, covered by non-keratinizing squamous epithelium. (C) From left to right: clinical appearance of the affected eye with pathological sampling; the submitted tissue surface covered by squamous epithelium; and presence of fibroadipose tissue, cartilage, and salivary gland tissue beneath.

Table 3 summarizes in detail the pathologic features of patients with corneal dermoid. Squamous epithelium coverage was present in all cases (100.0%; 95% CI: 97.0%-100.0%) (Figures 3A2, 2B2, 2C2). Proliferative fibrous tissue was found in 81.0% of patients (47/58; 95% CI: 70.6%-91.4%), and adipose tissue along with skin appendages was observed in 77.6% of cases (45/58; 95% CI: 66.5%-88.6%) (Figures 3A3, 3B3). Hyalinization was present in 41.4% of the patients (24/58; 95% CI: 28.3%-54.4%), while cartilage and salivary gland elements were rare, occurring in only 1.7% of cases (1/58; 95% CI: 0.0%-5.1%) (Figure 3C3). These findings provide a detailed overview of the pathological features associated with corneal dermoid, highlighting the common presence of squamous epithelium coverage, proliferative fibrous tissue, and adipose tissue with skin appendages, while hyalinization and cartilage or salivary gland elements are less frequently observed.

**Table 3:** Pathological characteristics of corneal dermoid patients

Pathological characteristics	Frequency	Percentage	95%CI
Squamous epithelium coverage	58/58	100.0%	97.0%-100.0%
Proliferative fibrous tissue	47/58	81.0%	70.6%-91.4%
Adipose tissue and skin Appendages	45/58	77.6%	66.5%-88.6%
Hyalinization	24/58	41.4%	28.3%-54.4%
Cartilage and salivary gland e	1/58	1.7%	0.0%-5.1%

Treatment methods

**Figure 4:**
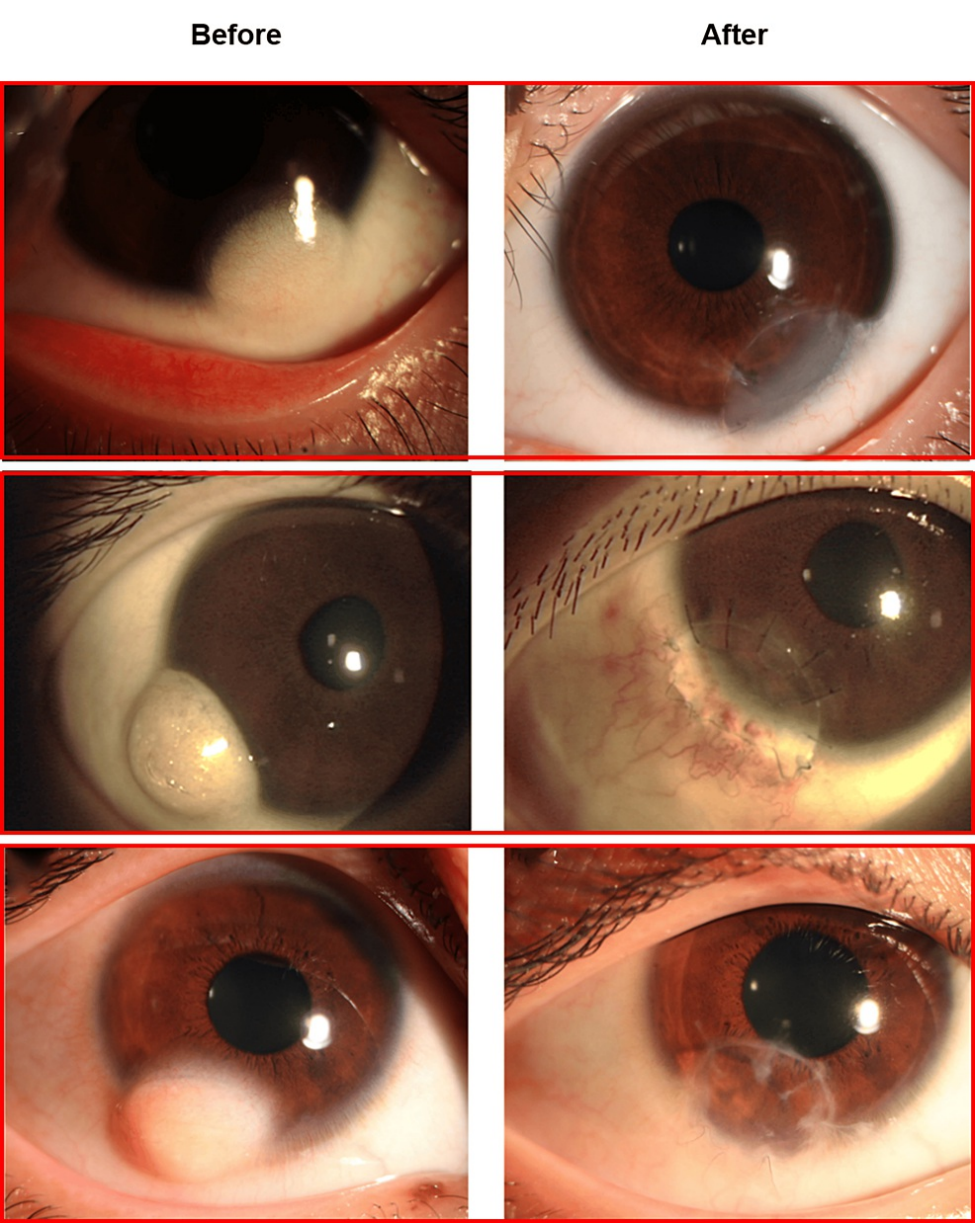
Appearance of a limbal dermoid before and after lamellar keratoplasty

**Figure 5:**
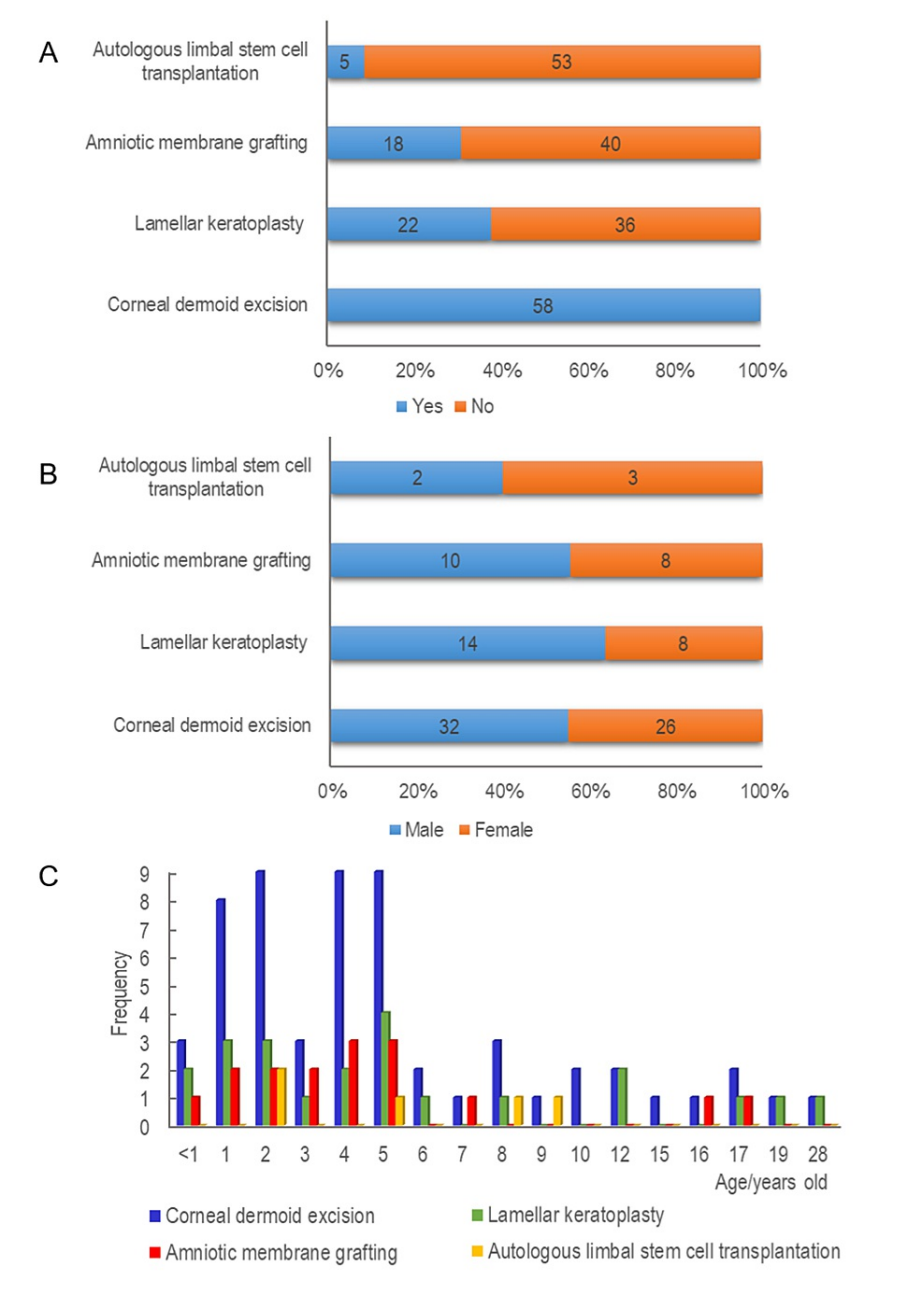
Age and gender distribution for different surgical methods (A) Distribution of different surgical methods; (B) gender distribution for different surgical methods; (C) age distribution for different surgical methods.

All 58 corneal dermoid patients underwent corneal dermoid excision. Figure 4 displays preoperative and postoperative images of the ocular surface in patients who received lamellar keratoplasty. Figure 5 presents the age and gender distribution for different surgical methods used in treating corneal dermoid. In Panel A, the distribution of surgical methods is shown. Corneal dermoid excision was performed in all 58 cases. Lamellar keratoplasty was conducted in 22 patients, amniotic membrane grafting in 18 patients, and autologous limbal stem cell transplantation in five patients. Panel B illustrates the gender distribution for these surgical methods. Among the 58 patients who underwent corneal dermoid excision, 32 were male and 26 were female. Lamellar keratoplasty was performed in 14 males and eight females. Amniotic membrane grafting was applied to 10 males and eight females. Autologous limbal stem cell transplantation was performed in two males and three females. Panel C depicts the age distribution for the different surgical methods. Corneal dermoid excision was most frequently performed in younger patients, particularly those under five years of age. Lamellar keratoplasty and amniotic membrane grafting were also more common in younger patients, whereas autologous limbal stem cell transplantation was less frequent and distributed across a wider age range. These findings provide insight into the demographics of patients receiving different surgical treatments for corneal dermoid, indicating a higher prevalence of procedures in younger patients and varied gender distributions across the surgical methods.

These findings highlight the age and gender trends associated with various surgical treatments for corneal dermoid. Lamellar keratoplasty and amniotic membrane grafting are predominantly performed on younger children, emphasizing the need for age-appropriate surgical planning. This is critical for optimizing surgical outcomes and planning future treatments. Further research with larger sample sizes is needed to validate these findings and explore additional factors that may influence treatment efficacy and patient recovery.

Treatment outcomes

During the follow-up period, 50 patients were monitored for at least six months postoperatively, with an average follow-up time of 12 months (range: 6-24 months). Eight patients were lost to follow-up. Among the 50 patients, there were no reports of corneal dermoid recurrence, indicating that surgical excision is potentially effective for the complete removal of corneal dermoid, with a low probability of short-term recurrence.

Visual acuity changes post-surgery were a primary observation metric during follow-up. Most patients showed improvement in visual acuity. Normal vision was restored in 29 patients (58.0%), including 20 males and nine females. Postoperative myopia or astigmatism occurred in 20 patients (40.0%). The prevalence of postoperative amblyopia was 20.0%. There was a statistically significant difference in the proportion of males and females with normal vision post-surgery (*χ*^2^=4.711, p=0.030). Postoperative visual impairment was observed in eight males (38.1%) and 13 females (61.9%), with 71.4% occurring in patients younger than five years and 71.4% affecting the right eye, primarily in patients with smaller tumors.

Among patients who underwent autologous limbal stem cell transplantation, all followed patients regained normal vision, except for one lost to follow-up. Among those who received amniotic membrane grafting, 10 patients (55.6%) regained normal vision, while eight patients (44.4%) reported varying degrees of visual impairment. Five patients who underwent lamellar keratoplasty were lost to follow-up; of the remaining, nine (52.9%) regained normal vision, and eight (47.1%) reported varying degrees of visual impairment. Postoperative visual outcomes are summarized in Table 4. Forty-eight patients did not report any discomfort symptoms during follow-up, while two patients reported an increased frequency of blinking in the affected eye post-surgery. This suggests that surgery generally does not cause long-term discomfort, and patients' quality of life is not significantly impacted.

**Table 4:** Postoperative visual outcomes in follow-up patients (n=50) ^a^There is a statistical difference in the prevalence of visual impairment between groups (*χ*^2^=4.711, p=0.030)

Variables	Category	Normal Vision, n (%)	Visual Impairment, n (%)	Myopia, n (%)	Astigmatism, n (%)	Amblyopia, n (%)	Hyperopia, n(%)
Total		29 (58.0)	21 (42.0)	5 (10.0)	16 (32.0)	10 (20.0)	2 (4.0)
Gender ^a^	Male	20 (69.0)	8 (38.1)	1 (20.0)	8 (50.0)	5 (50.0)	1 (50.0)
	Female	9 (31.0)	13 (61.9)	4 (80.0)	8 (50.0)	5 (50.0)	1 (50.0)
Age	0-5 years old	20 (69.0)	18 (85.7)	3 (60.0)	15 (93.7)	9 (90.0)	2 (100.0)
	6-10 years old	5 (17.2)	2 (9.5)	1 (20.0)	1 (6.3)	1 (10.0)	0 (0)
	>10 years old	4 (13.8)	1 (4.8)	1 (20.0)	0 (0)	0 (0)	0 (0)
Corneal invasion depth	<3 mm	18 (62.1)	13 (61.9)	3 (60.0)	10 (62.5)	6 (60.0)	0 (0)
	≥3 mm	11 (37.9)	8 (38.1)	2 (40.0)	6 (37.5)	4 (40.0)	2 (100.0)
Tumor surface	Smooth	6 (20.7)	8 (38.1)	1 (20.0)	7 (43.8)	5 (50.0)	0 (0)
	Uneven	23 (79.3)	13 (61.9)	4 (80.0)	9 (56.2)	5 (50.0)	2 (100.0)
Tumor area	≤20 mm^2^	16 (55.2)	11 (52.4)	3 (60.0)	7 (43.8)	4 (40.0)	1 (50.0)
	>20 mm^2^	13 (44.8)	10 (47.6)	2 (40.0)	9 (56.2)	6 (60.0)	1 (50.0)
Amniotic membrane grafting	Yes	10 (34.5)	8 (38.1)	2 (40.0)	7 (43.7)	4 (40.0)	0 (0)
	No	19 (65.5)	13 (61.9)	3 (60.0)	9 (56.3)	6 (60.0)	2 (100.0)
Lamellar keratoplasty	Yes	9 (31.0)	8 (38.1)	1 (20.0)	7 (43.7)	4 (40.0)	1 (50.0)
	No	20 (69.0)	13 (61.9)	4 (80.0)	9 (56.3)	6 (60.0)	1 (50.0)
Autologous limbal stem cell transplantation	Yes	4 (13.8)	0 (0)	0 (0)	0 (0)	0 (0)	0 (0)
	No	25 (86.2)	21 (100.0)	5 (100.0)	16 (100.0)	10 (100.0)	2 (100.0)

These findings highlight the overall positive outcomes of surgical treatments for corneal dermoid, with significant improvements in visual acuity and low recurrence rates. The statistical analyses and confidence intervals provide robust evidence supporting these outcomes, which are critical for informing clinical practice and optimizing patient care. Further research with larger sample sizes is needed to validate these findings and explore additional factors influencing treatment efficacy and long-term visual prognosis.

## Discussion

This retrospective study analyzed 58 corneal dermoid patients treated at our hospital from 2017 to 2021, focusing on clinical characteristics, treatment methods, and outcomes. Our analysis yielded the following key conclusions:

Clinical characteristics: First, corneal dermoid primarily occurs in pediatric and adolescent patients, with an average age of 6.3 years. This finding aligns with existing literature, suggesting that the disease is congenital and is typically detected at birth or in early childhood [5]. Although there were slightly more male than female patients, the gender difference was not significant, indicating no gender predisposition for corneal dermoid [7].

Lesion location: Second, more patients had lesions in the right eye than in the left, suggesting that special attention should be given to the right eye during diagnosis and examination. Tumors were primarily located at the temporal limbus, a potential high-incidence area for corneal dermoid, guiding clinical examinations. Additionally, bilateral dermoid was occasionally observed.

Tumor size and age correlation: Furthermore, the study found no significant correlation between tumor size, depth of corneal invasion, and patient age, which contrasts with literature stating that older patients often have larger tumors with deeper invasion [4]. Notably, some patients or their families reported that tumors gradually increased in size with age without significant vision loss. This phenomenon may result from timely surgical intervention at symptom onset in most patients, preventing further tumor growth and masking age-related growth. Additionally, the small sample size might have led to false-negative results, necessitating validation in larger populations in future research.

Pathological characteristics: Analyzing the collected results with complete pathological information reveals that corneal dermoid exhibits complex tissue origins and growth patterns, consistent with previous studies. Complete and typical pathological records feature squamous epithelium coverage, subepithelial fibrous tissue proliferation, adipose tissue, skin appendages, and hyalinization. However, rare glandular tissue was observed in one patient, underscoring the importance of considering diverse tissue components in diagnosing and treating corneal dermoid. Understanding these pathological characteristics is crucial for developing individualized treatment plans, selecting the most appropriate surgical methods, and maximizing treatment effectiveness while minimizing recurrence and complication risks.

Surgical outcomes: This study confirms the efficacy of surgical excision in preventing recurrence and improving visual outcomes for corneal dermoid, aligning with recent literature [8-11]. A significant number of patients (58%) achieved normal vision post surgery, but 40% developed myopia or astigmatism, highlighting the need for careful surgical planning and follow-up [11]. The 20% prevalence of postoperative amblyopia emphasizes the importance of early detection and treatment, especially in younger patients [10]. Gender differences in visual recovery, with females exhibiting higher rates of impairment, suggest a need for further investigation. Moreover, it should be emphasized that some visual impairments and their gender differences may not be caused by surgery. For example, some patients already had visual impairments before surgery. Surgical methods such as autologous limbal stem cell transplantation showed high success rates, while amniotic membrane grafting and lamellar keratoplasty had more varied outcomes, indicating a need for refined techniques [8,9]. Most patients reported no long-term discomfort, supporting the tolerability of these procedures. Future research should focus on optimizing surgical methods, addressing gender differences, and enhancing postoperative care to improve patient prognosis and quality of life.

Study limitations: This study has several limitations. First, as a single-center retrospective study, the sample size is relatively small and may not fully reflect all clinical characteristics and treatment outcomes of corneal dermoid. Therefore, future multicenter, large-sample studies are needed to validate these results further. Second, the data for this study were sourced from hospital medical records, which may be incomplete or subjective. Although measures were taken to ensure data accuracy and completeness, there is still a risk of bias. Future research should adopt more rigorous and systematic data collection methods to minimize the impact of incomplete and inaccurate information. Third, none of the cases in this study included imaging examinations, such as ultrasound biomicroscopy (UBM) to determine the depth and extent of the lesions, or gonioscopy to assess whether the lesions affected the anterior chamber structures [12]. Consequently, some important clinical information is lacking. Fourth, because of the limited number of cases and the variety of surgical methods, it is challenging to evaluate the effectiveness of different surgical approaches. The follow-up period was relatively short, limiting the evaluation of long-term treatment outcomes and distant complications. Future studies should extend the follow-up period to comprehensively assess the long-term efficacy and safety of various treatment modalities. Finally, this study did not adequately consider other factors that may influence treatment outcomes, such as the patient's overall health and immune status. These factors could significantly influence the treatment of corneal dermoid. Future research should further investigate these factors and include more variables in comprehensive analysis.

## Conclusions

This study provides a comprehensive retrospective analysis of 58 patients with corneal dermoid, revealing that it primarily affects children and adolescents, with the temporal limbus of the right eye being the most common site. The pathological features include squamous epithelium coverage, hair follicles, sebaceous glands, adipose tissue, and fibrous tissue. Surgical treatments were effective, with no recurrences and satisfactory structural and cosmetic outcomes, especially in female patients. The study contributes valuable data on the occurrence and treatment of corneal dermoid, supporting these surgical methods as effective. Future research should involve larger sample sizes and longer follow-up periods to uncover pathological mechanisms and improve treatments, enhancing patients' quality of life.
